# Age-related changes in physiology in individuals with lifetime bipolar disorder

**DOI:** 10.1192/bjo.2021.733

**Published:** 2021-06-18

**Authors:** Julian Mutz, Cathryn M Lewis

**Affiliations:** 1 Social, Genetic & Developmental Psychiatry Centre, Institute of Psychiatry, Psychology & Neuroscience, King's College London; 2 Social, Genetic & Developmental Psychiatry Centre, Institute of Psychiatry, Psychology & Neuroscience, King's College London, Department of Medical and Molecular Genetics, Faculty of Life Sciences & Medicine, King's College London

## Abstract

**Aims:**

Individuals with bipolar disorder have reduced life expectancy and may experience accelerated biological ageing. In individuals with lifetime bipolar disorder and healthy controls, we examined differences in age-related changes in physiology.

**Method:**

The UK Biobank study recruited >500,000 participants, aged 37–73 years, between 2006–2010. Generalised additive models were used to examine associations between age and grip strength, cardiovascular function, body composition, lung function and bone mineral density. Analyses were conducted separately in males and females with bipolar disorder compared to healthy controls.

**Result:**

Analytical samples included up to 272,462 adults (mean age = 56.04 years, SD = 8.15; 49.51% females). We found statistically significant differences between bipolar disorder cases and controls for grip strength, blood pressure, pulse rate and body composition, with standardised mean differences of up to -0.238 (95% CI -0.282 to -0.193). There was limited evidence of differences in lung function, heel bone mineral density or arterial stiffness. Case-control differences were most evident for age-related changes in cardiovascular function (in both sexes) and body composition (in females). These differences did not uniformly narrow or widen with age and differed by sex. For example, the difference in systolic blood pressure between male cases and controls was -1.3 mmHg at age 50 and widened to -4.7 mmHg at age 65. Diastolic blood pressure in female cases was 1.2 mmHg higher at age 40 and -1.2 mmHg lower at age 65.

**Conclusion:**

Differences in ageing trajectories between bipolar disorder cases and healthy controls were most evident for cardiovascular and body composition measures and differed by sex.

